# Reduced Blood Pressure Dipping Is A Risk Factor for the Progression of Chronic Kidney Disease in Children

**DOI:** 10.3390/biomedicines10092171

**Published:** 2022-09-02

**Authors:** Anna Deja, Piotr Skrzypczyk, Beata Leszczyńska, Małgorzata Pańczyk-Tomaszewska

**Affiliations:** 1 Department of Pediatrics and Nephrology, Doctoral School, Medical University of Warsaw, 02-091 Warsaw, Poland; 2 Department of Pediatrics and Nephrology, Medical University of Warsaw, 02-091 Warsaw, Poland

**Keywords:** chronic kidney disease, chronic kidney disease progression, estimated glomerular filtration rate, children, ambulatory blood pressure monitoring, blood pressure dipping

## Abstract

Background: Elevated blood pressure and proteinuria are well-established risk factors for chronic kidney disease (CKD) progression in children. This study aimed to analyze risk factors for CKD progress, emphasizing detailed ambulatory blood pressure (ABPM) data. Methods: In 55 children with CKD II–V, observed for ≥1 year or until initiation of kidney replacement therapy, we analyzed ABPM, clinical, and biochemical parameters. Results: At the beginning, the glomerular filtration rate (eGFR) was 66 (interquartile range—IQR: 42.8–75.3) mL/min/1.73 m^2^, and the observation period was 27 (16–36) months. The mean eGFR decline was 2.9 ± 5.7 mL/min/1.73 m^2^/year. eGFR decline correlated (*p* < 0.05) with age (r = 0.30), initial proteinuria (r = 0.31), nighttime systolic and mean blood pressure (r = 0.27, r = 0.29), and systolic and diastolic blood pressure dipping (r = −0.37, r = −0.29). There was no relation between mean arterial pressure during 24 h (MAP 24 h Z-score) and eGFR decline and no difference in eGFR decline between those with MAP 24 h < and ≥50 th percentile. In multivariate analysis, systolic blood pressure dipping (beta = −0.43), presence of proteinuria (beta = −0.35), and age (beta = 0.25) were predictors of eGFR decline. Conclusions: Systolic blood pressure dipping may be a valuable indicator of CKD progression in children.

## 1. Introduction

Chronic kidney disease (CKD) is an irreversible, progressive condition associated with impaired kidney structure or function. The initial, first stage of CKD is characterized by normal values of estimated glomerular filtration rate (eGFR), which gradually decrease with the progression of the disease, finally reaching the fifth, end stage of kidney disease (ESKD), associated with the necessity to initiate kidney replacement therapy (KRT) [1]. CKD in children is considered a rare disease, but the long life expectancy of the pediatric population and the potential prospects of a lifetime of KRT urge the need to prevent progression by all means necessary. Data from international registries indicate that mortality among children on dialysis has decreased over the years but remains elevated [2]. Noteworthy, multicenter registries report that cardiovascular disease is responsible for the largest percentage of deaths in patients on chronic dialysis [3,4].

Major efforts are put into slowing down the deterioration of kidney function, which is the primary goal of conservative treatment. Therefore, identifying potential risk factors for CKD progression is crucial in establishing the most accurate preventive methods for managing the disease. To date, numerous factors predicting CKD progression in children have been identified, including non-modifiable initial CKD stage, primary kidney disease, and age [5]. Hypertension and proteinuria are well-established, modifiable predictors of progression of kidney damage and decrease in eGFR in adults [6,7] and children [8].

The results of the multicenter ESCAPE (Effect of Strict Blood Pressure Control and ACE Inhibition on the Progression of CRF in Pediatric Patients) study showed that strict blood pressure control, defined as mean ambulatory blood pressure during 24 h (MAP 24 h) below the 50th percentile in ambulatory blood pressure monitoring (ABPM), significantly decreases risk for CKD progression and initiation of KRT [8]. Similarly, data from the Chronic Kidney Disease in Children (CKiD) cohort study revealed that lowering office blood pressure below the 90th percentile is associated with significantly slower CKD progression [9]. Based on the results of the ESCAPE study, Kidney Disease Improving Global Outcomes (KDIGO) recommends lowering blood pressure in all CKD children below the 50th MAP 24 h percentile, and, when an ABPM result is not accessible, using the 50th percentile of office blood pressure as a target value [10].

ABPM has been an established tool in assessing blood pressure in various pediatric populations for years. Routine ABPM blood pressure assessment in children with CKD is recommended by the European Society of Hypertension [11] and the American Heart Association [12]. ABPM blood pressure in children correlates better with hypertension-mediated organ damage (HMOD), compared to office and even home BP measurements [13]. In addition, it allows the assessment of parameters unavailable for office measurement, such as BP loads, nocturnal BP, circadian BP profile, BP variability, and even indirectly estimated arterial stiffness [12,14]. Of note, pediatric patients with CKD are prone to developing nighttime and masked hypertension, which also cannot be detected in office measurements [15].

Adult studies analyzed the influence of these ABPM parameters, e.g., abnormal circadian BP profile and nighttime hypertension, were risk factors for faster CKD progression [16,17], and 24 h diastolic blood pressure variability was associated with slower estimated glomerular filtration rate (eGFR) decline [18]. As no such data is available in children, we performed a study to evaluate the influence of different ABPM parameters on the CKD progression rate in children under our department’s care.

## 2. Materials and Methods

### 2.1. Study Group

**Figure 1:**
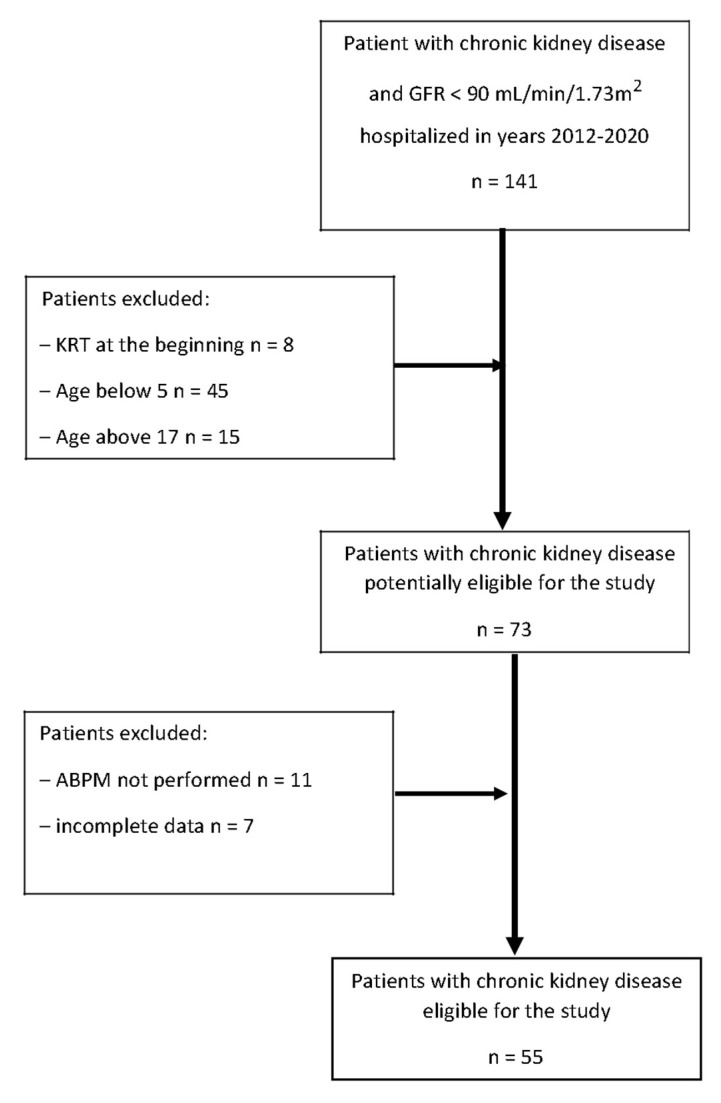
Flowchart of patients with chronic kidney disease hospitalized in the years 2012–2020 (GFR—glomerular filtration rate, KRT—kidney replacement therapy, ABPM—ambulatory blood pressure monitoring).

The study was a retrospective, single-center, observational analysis. Patients hospitalized in a tertiary care pediatric nephrology department between 2012 and 2020 were evaluated. The inclusion criteria were: diagnosed chronic kidney disease according to KDIGO guidelines [1], conservative treatment of CKD, eGFR < 90 mL/min/1.73 m^2^, and ABPM examination performed at the beginning of the observation period. The exclusion criteria were: kidney replacement therapy (KRT) at the beginning of the study, age below 5 and above 17 years, and lack of follow-up data. Fifty-five patients meeting the criteria were enrolled in the study and were observed for at least one year or until the initiation of KRT. The flowchart of patients is presented in Figure 1.

### 2.2. Baseline Clinical and Anthropometric Parameters

All initial examinations were performed simultaneously on the same day using a unified protocol. At the beginning of the study, we analyzed, based on available medical records clinical parameters: age (years), sex, and etiology of CKD according to the ESPN/ERA-EDTA (European Society for Paediatric Nephrology/European Renal Association-European Dialysis and Transplantation Association) registry [19], presence and value of proteinuria based on first urinary morning sample (mg/dL), and presence of arterial hypertension (AH) assessed following European Guidelines [11] and antihypertensive treatment; we also analyzed basic anthropometric parameters: height (cm), weight (kg), and body mass index (BMI) (kg/m^2^), expressed as Z-scores according to Polish normative values. Underweight was defined as BMI < 5th percentile, overweight as BMI between 85th and below 95th percentile, and obesity as BMI ≥ 95th percentile [20]. eGFR (mL/min/1.73 m^2^) was calculated using the Schwartz formula [21], based on serum creatinine concentration [mg/dL], assessed by the enzymatic method.

### 2.3. Ambulatory Blood Pressure Monitoring

We analyzed initial ABPM parameters in all included subjects. The 24 h blood pressure monitoring was performed using a SUNTECH OSCAR 2 device (SunTech Medical, Inc., Morrisville, NC, USA). All monitors were programmed to measure blood pressure every 15 min from 6 a.m. to 10 p.m. and every 30 min from 10 p.m. to 6 a.m. Periods of nighttime rest and daytime activity were determined by providing data in individual patient diaries. We measured systolic, diastolic, and mean blood pressures (SBP, DBP, MAP), heart rate (HR), blood pressure loads (BPL), and pulse pressure (PP) during 24 h as well as during activity (a) and rest (r) periods. Nocturnal systolic and diastolic blood pressure dipping (DIP sys, DIP dia) (%) were also evaluated. SBP, DBP, MAP, and HR were also expressed as Z-scores [22] and were interpreted using global standards for ABPM [12]. Disturbed circadian blood pressure profile was diagnosed when DIP sys or DIP dia was below 10% [12].

### 2.4. Follow-Up

After the observation period of at least one year or until initiation of KRT, we analyzed clinical parameters: duration of follow-up period (months), eGFR (mL/min/1.73 m^2^) [21], and CKD stage based on eGFR and KDIGO guidelines [1] as well as presence of arterial hypertension, antihypertensive treatment, and presence and range of proteinuria (urinary morning sample) (mg/dL). Annualized eGFR slope (mL/min/1.73 m^2^/year) was then calculated based on the total eGFR change and time of observation. Moreover, the time to deterioration of CKD by at least one stage following KDIGO [1] was assessed in each patient. One of the patients was lost to follow-up until they presented with end-stage kidney disease and, therefore, was excluded from analysis regarding CKD progression by one stage.

### 2.5. Endpoints

The primary endpoint was the annualized eGFR slope.

Secondary endpoints were progression to end-stage kidney disease (ESKD) requiring KRT, deterioration of CKD by at least one stage following KDIGO [1], and fast progression of CKD, defined by the ESCAPE clinical trial [8] as annualized eGFR decrease of ≥3 mL/min/1.73 m^2^/year.

### 2.6. Statistical Analysis

Collected data were anonymized and stored as a database with double coding (Excel 365, Microsoft 365, Microsoft, Redmond, WA, USA) and then statistically analyzed using Dell Statistica 13.3 PL software (TIBCO Software Inc., Palo Alto, CA, USA).

We assessed the normality of data using the Shapiro–Wilk test. Depending on the distribution, data are expressed as mean ± standard deviation (SD) for variables with normal distribution or median (interquartile range—IQR) for variables with distribution other than normal. The analysis required applying the following statistical tests: Mann–Whitney U test or Student’s t-test for independent groups, Spearman’s rank correlation or the Pearson correlation (where applicable, based on the normality of data), Student’s t-test or Wilcoxon test for dependent observations, Kruskal–Wallis ANOVA test, and chi-squared test. We also applied Kaplan–Meier survival analysis in regards to reaching secondary endpoints. Survival curves were compared using the F Cox test. Multivariate analysis was performed using the general regression and Cox proportional-hazard step-wise regression models. The variables were introduced into the model, excluding the ones that correlated with each other with r > 0.60 to avoid collinearity. The criterion for inclusion in the final model was *p* < 0.050, and *p* > 0.150 excluded the variable from the model. The results of multivariate analyses were expressed as beta, confidence interval (CI), *p*-value, and hazard ratio for Cox models. We also used receiver operating characteristics (ROC) analysis to calculate the area under the curves (AUC) and Youden’s index to identify the appropriate cut-off values of prognostic factors. The results were considered statistically significant, with *p* values < 0.050.

### 2.7. Ethical Issues

The researchers obtained approval from the local bioethics committee to conduct the study (approval no. AKBE/160/2022, 13 June 2022). All procedures involving human participants were in accordance with the highest ethical standards of the institutional research committee and were performed in accordance with the Declaration of Helsinki on the treatment of human subjects and its later amendments. According to Polish Act on Professions of Doctor and Dentist (Act of 5 December 1996 with later Amendments, Art. 21 Par. 4), formal informed consent was not required due to the retrospective nature of the study.

## 3. Results

### 3.1. Characteristics of the Examined Group

The initial clinical and anthropometric parameters of the examined group are shown in Table 1. In the studied group, the dominant sex was male. Most patients at the beginning of observation were in CKD stage II or III (46 children, 83.6%). Underweight was revealed in 2 patients (3.6%), overweight in 11 (20.0%), and obesity in 1 (1.8%). Congenital anomalies of the kidney and urinary tract (CAKUT) were the most common primary kidney diseases.

The median duration of observation was 27 (16–36) months. The minimal observation period was 6 months (initiation of KRT), and the maximal observation period was 95 months. Median eGFR at the end of observation was 60.1 (24.5–69.0) mL/min/1.73 m^2^/year. The mean annualized eGFR slope was 2.9 ± 5.7 mL/min/1.73 m^2^/year. In the course of the study, nine (16.4%) patients required initiation of KRT after the median of 25 (13–37) months. Kidney function deteriorated by at least one stage in 16 (29.1%) children. Twenty-four (43.6%) children were classified into a fast-progressing group based on annualized eGFR slope (≥3 mL/min/1.73 m^2^/year). The median time to deterioration of kidney function by ≥1 stage was 21 (6–31) months.

Table 2 displays the prevalence of well-established risk factors for CKD progression–the presence of proteinuria and hypertension—at the beginning of the study and after the observation period, as well as the use of antihypertensive medications. The most commonly administered medications were renin–angiotensin–aldosterone system blockers and calcium channel blockers.

### 3.2. Annualized eGFR Decrease

ABPM parameters and their correlations with annualized eGFR slope are displayed in Table 3. eGFR was correlated positively with systolic blood pressure and mean arterial pressure during rest (SBPr MAPr, respectively). We also found statistically significant negative correlations between eGFR slope and blood pressure dipping, both systolic and diastolic (DIP sys, DIP dia, respectively). There were no significant correlations between eGFR decline and other ABPM parameters.

A disturbed circadian profile of systolic blood pressure was found in 15 (27.3%) patients, 4 (7.3%) of whom also had a disturbed circadian profile of diastolic blood pressure. The remaining patients had a normal circadian blood pressure profile.

We also found significant positive correlations between eGFR slope and age (years) (r = 0.30, *p* = 0.026) as well as the initial proteinuria (r = 0.31, *p* = 0.020). There was no significant difference in eGFR slope regarding BMI Z-score (r = 0.03, *p* = 0.808) and the primary kidney disease (*p* = 0.429, Kruskal–Wallis ANOVA test).

In multivariate analysis, SBP dipping (%) (beta = −0.43, *p* < 0.001), presence of proteinuria (beta = −0.35, *p* = 0.004) and age (years) (beta = 0.25, *p* = 0.038) were the only predictors of eGFR decline.

### 3.3. Progression of CKD by at Least One Stage

**Figure 2:**
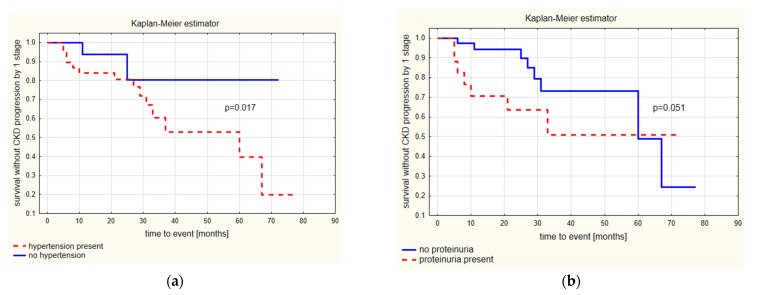
Kaplan–Meier estimator (F Cox test) for survival without CKD progression by ≥1 stage for children (**a**) with and without initially diagnosed arterial hypertension and (**b**) with and without initially present proteinuria.

In the Kaplan–Meier estimator, children with no arterial hypertension had a significantly longer survival time without CKD progression, by at least one stage, than children diagnosed with AH at baseline (*p* = 0.017), despite the treatment (Figure 2a). No such relationship was found for the presence of proteinuria (*p* = 0.051) (Figure 2b). There was no other significant predictor of progression of CKD, by at least one stage.

The Cox proportional-hazard step-wise regression model showed that only SBP dipping (%) (beta = −0.17, *p* = 0.024, hazard ratio 0.84, 95% CI 0.72–0.98) was a statistically significant risk factor for CKD progression by one stage.

### 3.4. Fast CKD Progression

**Figure 3:**
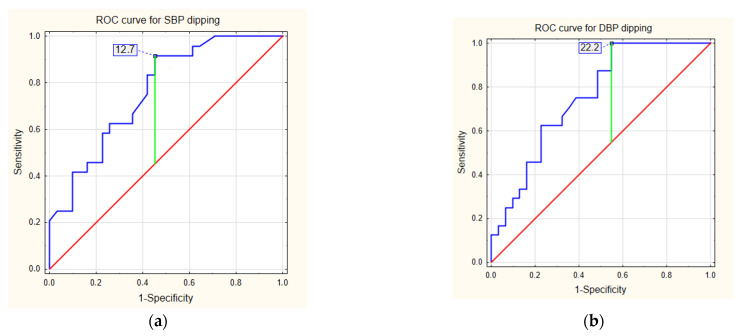

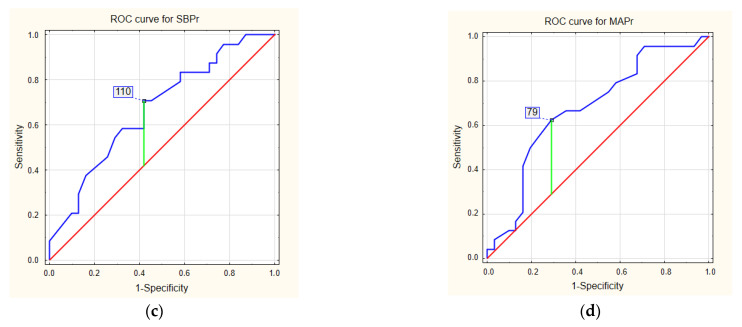
Receiver operating characteristic (ROC) curves for prognosing eGFR slope ≥ 3 mL/min/1.73 m^2^/year by destimulants: (**a**) SBP dipping and (**b**) DBP dipping; stimulants: (**c**) systolic blood pressure during rest and (**d**) mean arterial pressure during rest.

mlROC analysis results are presented in Table 4 and Figure 3. This analysis showed a statistically significant profile with high sensitivity for SBP and DBP dipping (%) as destimulants and SBPr and MAPr [mm Hg] as stimulants, in prognosing a fast decrease in eGFR.

### 3.5. KRT Initiation

We found no factors useful in prognosing the necessity of KRT initiation either in univariate or in multivariate models.

### 3.6. Mean Arterial Pressure

**Figure 4:**
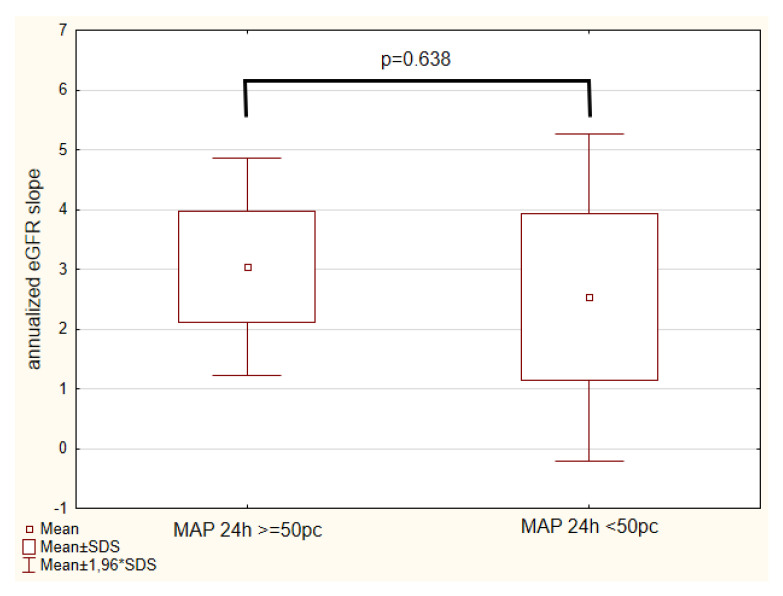
Annualized eGFR slope (mL/min/1.73 m^2^/year) in groups of children with MAP 24 h ≥ 50th percentile and MAP 24 h < 50th percentile. eGFR—estimated glomerular filtration rate, MAP—mean arterial pressure.

We found no significant correlation between annualized eGFR slope and mean arterial pressure z-score (MAP 24 h z-score). We divided the analyzed population into two groups: children with MAP 24 h ≥50th percentile (n = 41) and MAP 24 h < 50th percentile (n = 14). We found no significant difference in annualized eGFR slope between those groups (3.1 ± 5.9 vs. 2.5 ± 5.2 mL/min/1.73 m^2^, *p* = 0.638) (Figure 4).

## 4. Discussion

Numerous studies, mainly in adult patient populations, have identified risk factors for the fast progression of chronic kidney disease. Traditionally they are divided into non-modifiable and modifiable ones. The former include age, gender (in adults), race, cause of CKD, stage of CKD at the beginning of follow-up (CKD progression is logarithmic rather than linear) [5,6,7], or certain genetic polymorphisms, e.g., elements of the renin–angiotensin–aldosterone system or GSTM1 deletion, as revealed in CKiD study [23]. Modifiable risk factors include primarily proteinuria and arterial hypertension as well as uncompensated metabolic disorders: anemia, acidosis, and hyperphosphatemia [5,6,7].

Along with proteinuria, elevated blood pressure is the most significant factor in CKD progression. The prevalence of arterial hypertension in children with CKD is estimated to be approximately 50–60%, with the prevalence depending on the cause of CKD (more common in glomerular diseases and hemolytic uremic syndrome) and the stage of CKD (70% in children on dialysis) [9,24,25]. Hypertension in children with CKD is an essential factor for target-organ complications: left ventricular hypertrophy, thickening of the intima-media of the common carotid arteries, and increased arterial stiffness [26].

The most important consequence of arterial hypertension in the pediatric CKD population is its harmful impact on the progression of the disease, finally resulting in ESKD. The negative influence of elevated blood pressure was revealed in two multicenter studies—the European trial ESCAPE [8] and the American CKiD cohort [9]. Of note, the ESCAPE trial used ABPM and revealed that lowering BP below the 50th percentile of MAP is beneficial for slowing down eGFR loss. Later, this relationship was delineated in the CKiD cohort, which showed an association between high baseline [27] and time-updated blood pressure [9] and rapid eGFR decline.

Interestingly, our retrospective analysis did not demonstrate the significance of baseline blood pressure values in ABPM on the rate of CKD progression, beyond nocturnal systolic and mean blood pressure. The difference in results may have been derived firstly from a smaller group than in the cited ESCAPE and CKiD studies. In addition, unlike the ESCAPE study, we analyzed only initial ABPM—in the cited European study, ABPM was performed every six months. Moreover, initial eGFR was significantly higher in our group than in the ESCAPE study (68 vs. 46). The populations also differed in age (14.0 vs. 11.5) and etiology of CKD (CAKUT: 40% vs. 69%) [8].

Nighttime hypertension, including isolated nighttime hypertension, is common in adult and pediatric patients with impaired renal function. Mitsnefes et al. found nighttime hypertension was detected in 41% of pediatric CKD patients, attenuated systolic dipping in 59%, and diastolic dipping in 31% [28]. Sympathetic overdrive, endothelial dysfunction, and renin–angiotensin–aldosterone system activation are considered the most important pathogenetic mechanisms for the elevation of blood pressure during the night [29,30]. Nighttime hypertension and non-dipping circadian blood pressure pattern are risk factors for cardiovascular complications [31]. Nighttime hypertension (more precisely: masked uncontrolled nighttime hypertension) was a risk factor for left ventricular hypertrophy in adult Chinese patients with CKD [32]. In the analysis of patients from the 4C study, elevated night-time blood pressure was associated with increased markers of arterial damage and with left ventricular hypertrophy, and isolated nighttime hypertension was associated with thickening of common carotid artery intima-media thickness [33]. In the CKiD cohort, non-dipping was found in 41% of CKD children, though no statistically significant association between dipping and left ventricular mass index was revealed [34].

Adult data suggest that nighttime hypertension is also associated with a faster rate of CKD progression. Retrospective analysis of Chinese adult patients with non-dialysis CKD and arterial hypertension showed that masked uncontrolled hypertension is an independent risk factor for the composite kidney outcome, defined as ESKD or a 50% reduction in eGFR [32]. Similarly, a Japanese adult retrospective study showed that hypertensive non-dipper patients had the worst renal outcome. Conversely, the same group revealed that dipping status does not influence the risk for CKD progression in normotensive patients [16,35]. Wang et al. found in their prospective observation that the reverse dipping phenomenon was associated with the worst renal outcome, independent of 24 h systolic blood pressure values [17]. Despite these studies differing slightly in results and despite, arguably, the greater importance of non-dipping in hypertensive patients, the importance of analyzing the circadian pressure profile in all patients with CKD must be established. To the best of our knowledge, this is the first pediatric study to reveal that nighttime blood pressure is an independent marker of fast CKD progression. Although dipping status in children with CKD has been the subject of many analyses [28,33,34,36], its relationship with the rate of eGFR loss has not yet been evaluated.

The results of our retrospective study add a voice to the debate about the treatment of arterial hypertension in children with CKD. As mentioned in the introduction, current KDIGO guidelines recommend following 24 h MAP values from ABPM and lowering BP below the 50th percentile in all pediatric CKD patients. Notably, the authors base their recommendations only on the ESCAPE study [8]. It remains an open question whether such a standardized approach is the best therapeutic option for all patients, including those in the earlier stages of CKD. Indeed, the results of our study indicate the need to perform ABPM in children with chronic kidney disease and analyze the circadian blood pressure profile. Our study was not designed to demonstrate the beneficial effect of normalizing the diurnal profile in these patients. Prospective observational and interventional studies are needed to evaluate the impact of nocturnal BP normalization on the progression of CKD in children.

Proteinuria is a well-established risk factor for CKD progression or eGFR decline. Both CKiD [37] and the Italian registry (ITALKID) [38] revealed proteinuria as a significant, independent risk factor for rapid eGFR decline. Proteinuria is not only a symptom of kidney disease but also a risk factor for progression to kidney failure. The pathophysiology linking proteinuria to loss of kidney function is incompletely understood, but current hypotheses focus on the proximal tubular cell response to filtered protein [39]. The results of our study confirm what is known from the experimental data and numerous works on adults and the less numerous works on children. In our retrospective cohort, the rate of eGFR loss correlated with proteinuria, and the presence of proteinuria was a risk factor for CKD progression in multivariate analysis, indicating that it is difficult to identify the lower limit of harmful proteinuria clearly.

Age was another significant factor for CKD progression in our cohort. Similar findings were found in a retrospective analysis of North American Pediatric Renal Transplant Cooperative Study (NAPRTCS) patients [5] and the CKiD cohort [37]. Age is thought to significantly impact the natural course of CKD in children. In the first two years of life, there is a natural increase in eGFR resulting from a physiological increase in filtration rate in a single nephron, which is a consequence of increasing nephron volume and blood perfusion. Age less than five years (and, thus, inability to reliably interpret ABPM) was an exclusion criterion for our study. In contrast, during the teenage years, there is an acceleration in the rate of decline of eGFR in children, due to an increase in muscle mass and production of nitrogenous metabolic products [21,40].

The particular strength of our study is a unique, detailed analysis of ABPM parameters that, to the best of our knowledge, has not been performed in a pediatric cohort before. This analysis contributes new data and indicates potential new risk factors for the progression of CKD in children.

However, we are also aware of the limitations of the study. Firstly, the retrospective nature of the study prevented us from an analysis of other biochemical parameters (e.g., lipid and calcium–phosphorus metabolism parameters) due to incomplete data. The Oscar 2 SunTech ABPM device, although validated in adults, does not have a pediatric validation yet. However, it was found to be valuable and reliable in our numerous previous studies. Significantly, in our study, we correlated the rate of eGFR loss only with blood pressure values from the initial ABPM recording—the impact of blood pressure during the follow-up period was not analyzed. In our cohort, as already mentioned, the patients presented with relatively high initial eGFR. Proteinuria was assessed only using a single morning sample, not a well-established urinary protein/creatinine ratio or a 24 h collection. A single-center character of the study was associated with a relatively small number of patients, not diversified in terms of, e.g., race. Therefore, our further efforts will be focused on broadening the research into a multicenter study.

## 5. Conclusions

In our retrospective single-center study, we analyzed the effect of various ABPM blood pressure parameters on the rate of CKD progression in children. In our cohort, in addition to proteinuria and age, the progression of CKD was dependent on systolic and mean resting blood pressure and systolic and diastolic blood pressure dipping. Unlike in the ESCAPE study, there was no association between the rate of CKD progression and 24 h mean arterial pressure (ABPM MAP 24 h). Despite the differences with the latter trial (e.g., less advanced stage of CKD) and the limitations of our study, we believe that when assessing blood pressure in children with CKD, close attention should be paid to the nocturnal blood pressure and circadian blood pressure profile. Large multicenter studies are needed to demonstrate the benefit of normalizing the nocturnal blood pressure and circadian profile, e.g., by giving antihypertensive drugs in the evening.

## Figures and Tables

**Table 1:** Clinical and anthropometric parameters at the beginning of the observation period.

Parameter	Initial Values	*p*
Boys/girls	38/17	0.005
Age (years)	14.0 (9.9–15.6)	-
eGFR (mL/min/1.73 m^2^)	66.0 (42.8–75.3)	-
Height Z-score	−0.2 ± 1.3	-
Weight Z-score	0.3 (−0.4–0.8)	
BMI Z-score	0.4 (−0.2–0.9)	-
Etiology of CKD (n, %)		
CAKUT	22 (40.0%)	<0.001
Hereditary diseases	12 (21.8%)	
AKI (HUS included)	9 (16.4%)	
Glomerular diseases	7 (12.7%)	
Cystic kidney diseases	3 (5.5%)	
Other *	2 (3.6%)	

eGFR—estimated glomerular filtration rate, BMI—body mass index, CKD—chronic kidney disease, CAKUT—congenital anomalies of the kidney and urinary tract, AKI—acute kidney injury, HUS—hemolytic uremic syndrome. * included: post-Ewing sarcoma Fanconi syndrome (n = 1), chronic tubulointerstitial nephritis associated with mesalazine treatment (n = 1).

**Table 2:** Incidence of hypertension and proteinuria at the beginning and after observation.

	Initial	After Follow-Up
Presence of hypertension (n, %)	38 (69.1%)	40 (72.7%)
Antihypertensive medications		
Ca blockers	23	28
ACEI/ARB	24	34
Beta blockers	6	10
Alpha blockers	4	5
Diuretics	2	2
Presence of proteinuria (n, %)	18 (32.7%) 131.5	22 (40.0%) 104.5
Proteinuria (mg/dL)	(36.5–202.3)	(49.8–212.8)

ACEI—angiotensin-converting enzyme inhibitors, ARB—angiotensin II receptor blockers.

**Table 3:** Values of ABPM parameter measurements and their correlations with eGFR slope.

ABPM Parameter	Measurement [Mean ± SD/Median (IQR)]	Correlation with eGFR Slope *	
		R	*p*
SBP 24 h (mm Hg)	120.9 ± 8.7	0.11	0.433
SBP 24 h z-score	1.3 ± 1.3	0.01	0.966
DBP 24 h (mm Hg)	69.6 ± 7.9	0.14	0.317
DBP 24 h z-score	0.4 ± 1.4	0.11	0.442
MAP 24 h (mm Hg)	86.9 ± 7.4	0.14	0.320
MAP 24 h z-score	1.0 ± 1.3	0.05	0.739
SBPL 24 h (%)	23.0 (10.0–44.0)	0.02	0.866
DBPL 24 h (%)	12.0 (5.0–35.0)	0.02	0.911
PP 24 h (mm Hg)	51.2 ± 7.8	−0.02	0.913
HR 24 h (bpm)	81.2 ± 12.0	−0.13	0.350
HR 24 h z-score	−0.3 ± 1.2	−0.07	0.634
SBPa (mm Hg)	124.7 ± 8.9	0.05	0.705
SBPa z-score	1.0 ± 1.2	−0.06	0.671
DBPa (mm Hg)	72.0 (66.0–78.0)	0.05	0.731
DBPa z-score	0.0 (−1.9–1.0)	0.04	0.752
MAPa (mm Hg)	90.3 ± 7.7	0.11	0.422
MAPa z-score	0.7 ± 1.2	0.04	0.762
PPa (mm Hg)	51.0 ± 47.0	0.04	0.753
HRa (bpm)	85.0 ± 77.0	−0.14	0.298
HRa z-score	−0.8 ± 1.3	−0.01	0.954
SBPLa (%)	22.0 (8.0–46.0)	−0.05	0.743
DBPLa (%)	12.0 (3.0–30.0)	−0.03	0.822
**SBPr** (mm Hg)	**110.0 ± 8.8**	**0.27**	**0.045**
SBPr z-score	1.2 ± 1.2	0.15	0.268
DBPr (mm Hg)	60.2 ± 8.0	0.26	0.057
DBPr z-score	0.7 ± 1.5	0.24	0.074
**MAPr** (mm Hg)	**76.8 ± 7.4**	**0.29**	**0.032**
MAPr z-score	0.9 ± 1.2	0.26	0.056
PPr (mm Hg)	50.0 ± 45.0	0.10	0.449
HRr (bpm)	70.4 ± 10.9	−0.12	0.376
HRr z-score	0.0 ± 1.0	−0.09	0.522
SBPLr (%)	25.0 (0.0–53.0)	0.15	0.275
DBPLr (%)	12.0 (0.0–45.0)	0.12	0.368
**DIP sys (%)**	**11.8 ± 4.2**	**−0.37**	**0.006**
**DIP dia (%)**	**17.5 ± 6.6**	**−0.29**	**0.034**

* Pearson or Spearman’s rank correlations where applicable. SBP—systolic blood pressure, DBP—diastolic blood pressure, MAP—mean arterial pressure, PP—pulse pressure, HR—heart rate, bpm—beats per minute, L—load, a—during activity, r—during rest, DIP sys—systolic blood pressure dipping, DIP dia—diastolic blood pressure dipping. Bold indicates a statistically significant correlation (*p* < 0.050).

**Table 4:** Diagnostic accuracy of predicting annualized eGFR slope ≥ 3 mL/min/1.73 m^2^/year. SBP and DBP dipping acting as destimulants and DBPr and MAPr acting as stimulants.

Parameter	AUC (95% CI)	*p*	Cut-Off Value	Sensitivity (%)	Specificity (%)
DIP sys (%)	0.764 (0.641–0.887)	<0.001	12.7	91.1	54.8
DIP dia (%)	0.754 (0.628–0.880)	<0.001	22.2	100	45.2
SBPr (mm Hg)	0.671 (0.528–0.813)	0.019	110	70.8	58.1
MAPr (mm Hg)	0.677 (0.533–0.822)	0.016	79	62.5	71.0

AUC—area under the curve, CI—confidence interval, DIP sys—SBP dipping, DIP dia—DBP dipping, SBPr—systolic blood pressure during rest, MAPr—mean arterial pressure during rest.

## Data Availability

Data included in this study are available as Supplementary Materials.

## Supplementary Materials

The following are available online at https://www.mdpi.com/article/10.3390/biomedicines10092171/s1. Table S1: Patient data (patient_data.xlsx).
